# Analytical Insights into Protein–Alum Interactions and Their Impact on Conformational Epitope

**DOI:** 10.3390/pharmaceutics16030420

**Published:** 2024-03-19

**Authors:** Alessio Corrado, Mila Toppazzini, Alessandro Vadi, Carmine Malzone, Rosy Galasso, Alessandro Donati, Riccardo De Ricco, Francesco Berti

**Affiliations:** 1 GSK, Vaccines Srl, via Fiorentina 1, 53100 Siena, Italy; alessio.x.corrado@gsk.com (A.C.); mila.x.toppazzini@gsk.com (M.T.); alessandro.x.vadi@gsk.com (A.V.); carmine.x.malzone@gsk.com (C.M.); rosy.x.galasso@gsk.com (R.G.); riccardo.x.de-ricco@gsk.com (R.D.R.); 2 Department of Biotechnology, Chemistry and Pharmacy, University of Siena, via A. Moro 2, 53100 Siena, Italy; alessandro.donati@unisi.it

**Keywords:** bacterial vaccines, adjuvant, protein subunit, vesicles, meningococcal

## Abstract

Several alum-adjuvanted vaccines have been licensed in the past 40 years. Despite its extensive and continuous use, the immune mechanism of action of alum adjuvants is not yet completely understood. Many different variables during the formulation process have been assessed as critical for alum-adjuvanted vaccines, although most of them are still not yet fully understood. The absence of a clear understanding of all the possible variables regulating the mechanism of action and the behavior that alum adjuvant imposes on the protein antigen may also be related to analytical challenges. For this reason, there is an urgent need for a fast and simple tool that is possible without a preliminary sample manipulation and is able to control the amount and the degree of antigen adsorption levels and their consistency across different production processes. This work attempts to develop new analytical tools with the aim of directly quantifying and assessing both the content and/or the purity of formulated alum-adsorbed antigens, without any preliminary sample manipulation (e.g., antigen desorption) being reported. In addition, the different confirmation/behavior in terms of the response to specific monoclonal antibodies in the presence of different ratios of alum-OH adsorbent antigens have been investigated. As a proxy to develop new analytical tools, three recombinant protein adsorbed models were used as follows: Neisseria adhesin A (NadA), Neisserial Heparin Binding Antigen (NHBA), and factor H binding protein (fHbp) as antigens, as well as aluminum hydroxide (AH) as an adjuvant system. The selection of the adjuvanted system model was dictated due to the substantial quantity of the literature regarding the protein structure and immunological activities, meaning that they are well characterized, including their adhesion rate to alum. In conclusion, three different analytical tools were explored to quantify, detect, and study the behavior of antigens in the presence of the alum adjuvant.

## 1. Introduction

Over the past century, vaccination has played a crucial role in the reduction in death rates and disease caused by infectious diseases, and it has been estimated that vaccines save at least 2–3 million lives per year worldwide [1]. In vaccine development, a key role is played by molecules that enhance the immune response of the antigen without significantly increasing the toxicity or reactogenicity of the vaccine, and they are known as adjuvants. Adjuvants are molecules that boost the effectiveness and the persistence of a vaccine antigen’s immune response without a significant increase in toxicity or reactogenicity [2].

The adjuvant activity of aluminum-containing compounds was first discovered in 1926 when an alum-precipitated diphtheria vaccine showed improved antigenic properties compared to those of a standard diphtheria vaccine [3,4]. Since then, aluminum-containing compounds have been routinely used in vaccine formulations due to their good record of safety, low cost, and compatibility with various antigen classes [4,5]. Two types of aluminum-based adjuvants are commonly used in licensed vaccines as follows: (i) aluminum hydroxide (aluminum oxyhydroxide or alum) and (ii) aluminum phosphate (aluminum hydroxy phosphate or alum-P). Alum only has hydroxyl groups on its surface and appears as a crystalline structure composed of needle-shaped nanoparticles, which tend to aggregate up to an average diameter of 10 µm. In the vaccine formulation, at a neutral pH, alum has a positive charge due to its point of zero charge (PZC) of 11.4. This is in contrast with alum-P, which has both hydroxyl and phosphate groups on its surface in a ratio that depends on the manufacturing conditions. The amorphous structure of alum-P is due to particles of around 50 nm, which cause irregular aggregates. At a neutral pH, it has a negative charge due to its PZC being between 4.5 and 5.5. These differences between alum and alum-P affect the adsorption properties and the behavior of antigens on its surface [6,7].

Since 1977, the World Health Organization (WHO) has recommended an antigen adsorption level of ≥80% for vaccines containing aluminum adjuvants [8,9,10], highlighting the importance of controlling the degree of antigen adsorption levels and their consistency across different production processes [11].

Despite its extensive and continuous use, the immune mechanism of the action of aluminum-containing adjuvants is not yet completely understood [12,13]. In addition, from a technical point of view, many different variables during the formulation process have also been assessed as critical for alum-adjuvanted vaccines, although most of them are still not yet fully understood. For example, Laera et al. [14] have recently reported that for the preparation of an alum-based tetravalent protein vaccine, the formulation strategy (sequential, competitive, and separate) plays an important role in the final antigen distribution across alum particles over time, which is related to the electrostatic strength of each antigen. In addition, Kumru et al. showed that properties such as particle size, point of zero charge (PZC), and alum surface area also influence the adsorption of vaccine antigens [15].

The absence of a clear understanding regarding the mechanism of action and the behavior of alum-containing adjuvants is in part due to analytical challenges. The determination of the purity, potency, and content of the antigens in alum-formulated products, without sample manipulation, is difficult due to complex formulations, low antigen doses, and the presence of colloidal systems, amongst other factors; therefore, to characterize the antigens in adjuvanted vaccines, it is necessary to desorb and recover them completely from the adjuvant surface [16]. The high concentration of salts and surfactants used in the desorption procedure may influence some chemical and physical properties of the product, which may in turn compromise its integrity and alter its behavior. Furthermore, some components of the desorption buffer may remain in the aqueous phase of the sample and potentially cause interference with the assay (e.g., surfactants impact high-performance liquid chromatography (HPLC) methods, and histidine buffers and sucrose are not suitable for some colorimetric assays). The understanding of the physico-chemical proprieties of the two components in the formulation (adjuvant–antigen) is crucial. Furthermore, critical parameters such as the degree of antigen desorption and colloidal stability must be monitored over time since they could have an impact on the efficacy, safety, and shelf life of the final product.

For this reason, there is an urgent need for a fast and simple tool that is possible without preliminary sample manipulation and is able to control the amount and the degree of antigen adsorption levels and their consistency across different production processes. Herein, we report attempts to develop new analytical tools with the aim of directly quantifying and assess both the content and/or the purity of formulated alum-adsorbed antigens, without any preliminary sample manipulation (e.g., antigen desorption). In addition, the different conformation/behavior in terms of response to specific monoclonal antibodies (mAbs) in presence of different ratios of alum-OH adsorbent antigens have been investigated. As a proxy to develop new analytical tools, three model adsorbed recombinant proteins were used: Neisseria adhesin A (NadA), Neisserial Heparin Binding Antigen (NHBA), and factor H binding protein (fHbp) as antigens and alum-OH as an adjuvant system (Figure S1). The selection of the adjuvanted system model was dictated by the substantial quantity of studies in the literature regarding protein structure and immunological activities, meaning that they are well characterized, including their adhesion rate to aluminum [17,18,19,20].

## 2. Materials and Methods

### 2.1. Materials Used in the Capillary Electrophoresys (CE)

The buffers were prepared using the following materials: Tris(hydroxymethyl)aminomethane, Sodiumdodecylsulfate (SDS), Methanol, and Histidine purchased from Sigma Aldrich (Merck KGaA, Darmstadt, Germany). Hydrochloric acid with fuming 37% HCl was purchased from Merck KGaA, Darmstadt, Germany; Hydrogen Peroxide was purchased from GE Healthcare (London, UK). Sodium Hydroxide, 50%(*w*/*w*) (NaOH) was purchased from J.T. Baker (Merck KGaA, Darmstadt, Germany). All the reagents were stored in accordance with manufacturer recommendations and used without further purification.

BGE (background electrolyte) solutions:

▪ To prepare the Tris Acetate buffers, a proper weight of Tris(hydroxymethyl)aminomethane was dissolved in ultra-purified water to reach the final desiderated molarity. A proper volume of glacial acetic acid was added to obtain the desired pH.
▪ To prepare the Tris Acetate SDS buffers, proper weights of Tris(hydroxymethyl)aminomethane and SDS were dissolved in ultra-purified water to reach the final desiderated molarity. A proper volume of glacial acetic acid was added to obtain the desired pH.

All the reagents were stored in accordance with manufacturer recommendations and used without further purification.

### 2.2. Antigens and Adjuvants

The alum adjuvant was obtained from GSK Vaccines (Marburg, Germany). The vaccine recombinant proteins were obtained from GSK Vaccines (Siena, Italy). fHbp fused with GNA2091, NadA, and NHBA fused with GNA1030 were used for the study. The isoelectric points (pI) were theoretically calculated as 4.6, 5.1, and 9.0, respectively.

#### 2.2.1. Preparation of Antigen Formulation without Alum

The antigen formulation solution was a mixture of the three recombinant proteins NHBA-NUbp, FHbp-GNA2091, and NadA.

The solutions were formulated freshly by diluting the proper volume of the NHBA-NUbp, FHbp-GNA2091, NadA, and drug substances in bulk (stored in aliquots at −20 °C) to reach the final composition: 0.1 mg/mL of each in ultra-purified water.

While the single-antigen solution without alum was freshly formulated by diluting the proper volume of each antigen, for NHBA-NUbp, FHbp-GNA2091, and NadA, the drugs were prepared in bulk (stored in aliquots at −20 °C) to achieve the final composition of 0.3 mg/mL of each in ultra-purified water.

#### 2.2.2. Preparation of Alum Samples

The alum samples were prepared by diluting of proper volume of aluminum hydroxide in bulk to reach the desired concentration of 3.0 mg/mL.

The single-antigen formulations with alum were formulated using the same protocol for each antigen. For the fresh formulation of the selected antigens (NHBA-NUbp, FHbp-GNA2091, NadA), dilution of the drug substances in bulk (stored in aliquots at −20 °C) to reach the fixed antigen concentration of 0.3 mg/mL was performed using proper volumes of alum stock solution to reach different alum concentrations (0.3, 0.5, 1, 2, 3.0 mg/mL) in ultra-purified water. The solutions were stirred for a minimum of 2 h to allow for protein adsorption on the alum.

### 2.3. Instrument and Separation Methods

The experiments were performed with a high-performance capillary electrophoresis system: PA800plus (AB Sciex, Framingham, MA, USA). Two different capillaries were used during the experiments:

▪ Uncoated fused silica column (AB Sciex, Framingham, MA, USA); inner diameter (ID): 50 μm; total capillary length: 70.2 cm (60 cm to detector).
▪ Neutral capillary linear polyacrylamide (LPA) coated capillary (AB Sciex, Framingham, MA, USA) with an inner diameter (ID): 50 µm; total capillary length: 50.2 cm (40 cm to detector).

A PDA with wavelengths set at 200 nm, 220 nm, and 280 nm was used as the detector and the data were acquired and elaborated upon using 32-Karat V10.1 software.

Before each run, the neutral capillary was sequentially rinsed for 5 min with water at 50 psi and 6 min with the selected assay BGE. The samples were loaded into individual microvials in an autosampler. The samples were injected in the capillary for 60 s at 0.5 psi. The separation step was performed for 60 min by applying 12 kV constant voltage in reversed polarity plus 0.2 psi.

Before each run, the uncoated fused silica capillary was sequentially rinsed for 3 min with water, for 3 min with a 30% MeOH solution, for 3 min with 0.1 M NaOH, and for 4 min with selected assay buffer, applying 10 psi for each step. The samples were loaded into individual microvials in an autosampler and were hydrodynamically injected into the capillary for 0.15 s at 0.5 psi. The separation step was performed by applying 15 kV constant voltage in normal polarity for 35 min.

The universal plastic vials for capillary electrophoresis and universal rubber vial caps, blue, were purchased from AB Sciex, Framingham, MA, USA.

### 2.4. Materials for In Vitro Relative Potency Assay (IVRP)

#### 2.4.1. Monovalent Formulations

The alum monovalent solutions were formulated by dilution of the proper volume of the NHBA-drug substance in bulk (stored in aliquots at −20 °C) to reach the final composition: 100 μg/mL NHBA in 10 mM L-histidine pH 6.3 buffer plus 6.25 mg/mL NaCl, 2% (*w*/*v*) sucrose. The alum concentration was varied from 0.5 to 3.0 mg/mL as the target (0.5, 1.0, 2.0, 3.0 mg/mL). The monovalent, freshly formulated at 3.0 mg/mL, alum was used as a reference when compared to monovalent formulations at different alum concentrations.

#### 2.4.2. Multivalent Formulations

The alum multivalent solutions were formulated freshly by dilution of the proper volume of each of the recombinant proteins NHBA-fHbp and NadA in bulk (stored in aliquots at −20 °C) to reach the final composition: 100 μg/mL of each recombinant protein in a 10 mM L-histidine pH 6.3 buffer plus 6.25 mg/mL NaCl, 2% (*w*/*v*) sucrose; the alum concentration was varied from 0.5 to 3 mg/mL as the target (0.5, 1.0, 2.0, 3.0 mg/mL). The multivalent sample formulated at 3.0 mg/mL of alum was used as a reference when compared to the multivalent or monovalent formulates at different alum concentrations.

#### 2.4.3. Antibodies

The monoclonal antibodies (mAbs) reported in Table 1 were provided by the GSK Immunoassay group. Phycoerythrin-conjugated anti-mouse secondary antibody (Jackson ImmunoResearch Laboratories, Inc., West Grove, PA, USA), diluted 1:400, was used as the secondary detection reagent.

### 2.5. Luminex Assay Procedure

**Figure 1:**
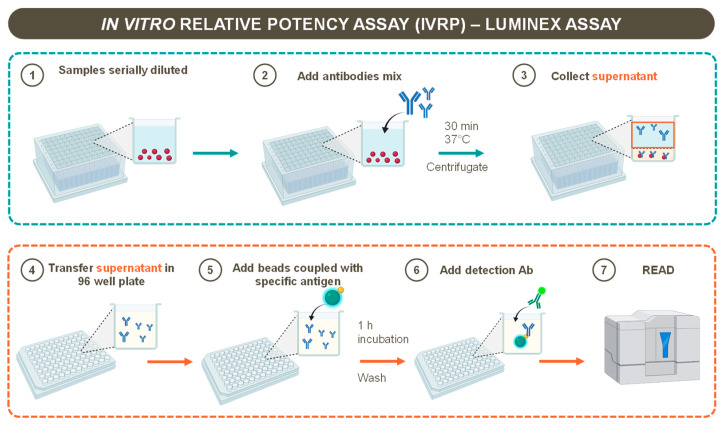
Schematic representation of IVRP-Luminex workflow.

The three recombinant proteins (NadA and fHbp, 40 µg/mL each, and NHBA, 80 µg/mL) were coupled to the carboxyl groups of 2.5 × 10^6^ MagPlex microspheres (Luminex Corporation, Austin, TX, USA) following the manufacturer instructions. Each antigen was coupled to a microsphere set, identifiable through its unique spectral signature. The samples were diluted 1:4 in assay buffer (PBS 1% Candor 0.05% Tween20) and 2 mL per well was transferred (600 μL/w) to a 96-well deep-well plate. A serial two-step dilution (300 μL/w) was performed in the assay buffer for eight points, and mAbs at working dilution were added in each well (300 μL/w). After a 30 min incubation step at 37 °C, the plates were centrifuged for 20 min at 1000× *g* acceleration 9 brake 3. The supernatant, containing the unbound mAbs (100 μL/w), was collected and transferred to 96-well flat-bottom plates, while the alum-antigen-mAbs complex remained on the well bottom. Following this step, an Ag-conjugated beads mix was added in each well and incubated for 60 min at room temperature on a shaker plate set to 150–160 rpm/min. Following incubation, the plates were washed with PBS using an automatized magnetic washer, HydroSpeed 96i (Tecan, Männerdorf, Switzerland). R-Phycoerythrin affine pure F (Ab) 2 fragment goat anti-mouse IgG-PE (Li StarFish cat n° 115-116-072) diluted 1:100 in PBS was used for detection in a 30 min incubation step at room temperature, on shaker plate settled at 150–160 rpm/min. Afterwards, the plates were washed with 1x PBS in by an automatized magnetic washer HydroSpeed 96i (Tecan, Männerdorf, Switzerland). The resulting unreacted mAbs complexed with beads–antigen were resuspended in 1x PBS (100 μL/w) and analyzed using the Luminex LX-200 system. The procedure was schematized in Figure 1.

### 2.6. Hydroxyl Radical Footprinting (HRF) Assay

#### 2.6.1. Materials 

Fe(NH_4_)_2_(SO_4_)_2_ (Ammonium iron(II) sulfate hexahydrate), tris(2-carboxyethyl)phosphine (TCEP), and sodium ascorbate were purchased from Sigma (Merck KGaA, Darmstadt, Germany. Na_2_HPO_4_, EDTA (ethylenediaminetetraacetate), and formic acid (FA) were purchased from Merck Millipore. The thiourea was purchased from Agilent Technologies (Santa Clara, CA, USA). H_2_O_2_ was purchased from GE Healthcare (Chicago, IL, USA). The Oasis HLB 1 cc cartridges were purchased from Waters (Milford, MA, USA). All the reagents were stored in accordance with manufacturer recommendation and used without further purification.

#### 2.6.2. Mass Spectra Acquisition

The mass spectra were acquired in resolution mode (*m*/*z* 300–1600) on a Thermo Fisher Scientific (Waltham, MA USA) Q Exactive Plus mass spectrometer equipped with a Heated Electrospray Ionization source (HESI-II). The MS data were acquired in positive mode using a data-dependent acquisition (DDA) dynamically choosing the five most abundant precursor ions (Top 5) from the survey scan at 70,000 resolutions. Fragmentation for peptide identification was obtained by higher-energy collisional dissociation (HCD) at 17,000 resolution and normalized collision energy (NCE) 26 eV. The automatic gain control (AGC) was set at 3 × 10^6^ for precursor ions and at 10^5^ for MS/MS acquisition; the isolation of precursor ions was performed with a 3 *m*/*z* window and isolation offset of 1 *m*/*z*. The maximum injection time was set at 200 msec for precursor ion acquisition and at 150 msec for MS/MS acquisition. The mass accuracy was ensured by monitoring the environmental contaminant Polysiloxane at 445.120025 *m*/*z* during the analysis.

#### 2.6.3. UPLC (Ultra-Performance Liquid Chromatography) Chromatographic Method

The chromatographic separation was performed using a C18-reversed phase column Acquity UPLC peptide CSH C18 130 Å, 1.7 µm 1 × 150 mm with a 60 min linear gradient of 28–85% buffer B (0.1% (*v*/*v*) formic acid (FA) in Acetonitrile (ACN)) at a flow rate of 50 μL/min and 50 °C column temperature on an Acquity I-Class UPLC (Waters).

#### 2.6.4. HRF Sample Preparation Protocols

**Figure 2:**
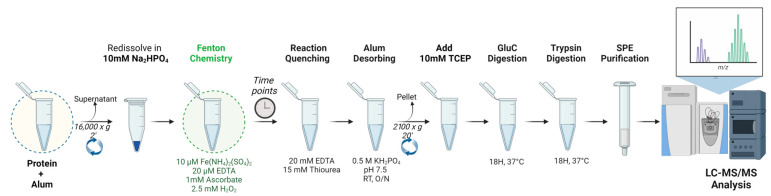
HRF schematic protocol workflow for alum-compound.

The hydroxyl radical footprinting (HRF) protocol was applied at same time on the four NHBA drug substances formulated at different alum concentrations (0.5, 1, 2, 3 mg/mL). For each formulation, the experimental workflow shown in the schematic procedure in Figure 2 was performed.

First, 4 mL of each alum formulation was centrifuged at 16,000× *g* for 2 min and the supernatant was removed. The pellet was redissolved in 500 µL of Na_2_HPO_4_ and split in 4 aliquots of 100 µL each. Fenton chemistry was performed by adding, in order, 10 µM Fe(NH_4_)_2_(SO_4_)_2_ with 20 µM EDTA, 1 mM sodium ascorbate, and 2.5 mM hydrogen peroxide mixed with 100 µL of the sample solutions previously obtained. Four aliquots of each sample were produced in order to replicate the reaction at 0, 2, 6, and 8 min time points (To 0 min treated samples, only Fe(NH_4_)_2_(SO_4_)_2_, EDTA, and ascorbate were added). After the reaction was completed, 20 µL of stop solution at 30 mM (EDTA and 15 mM thiourea) was added to the samples. The desorption process was then carried out by adding 0.5 M KH_2_PO_4_ pH 7.5 to each sample and leaving the samples stirring overnight at room temperature. The samples were centrifuged at 2100× *g* per 20 min, the supernatant was recovered, and the pellet was discharged. To the sample was then added TCEP to a final concentration of 15 mM to stabilize the oxidized residues after Fenton chemistry reaction. Double enzymatic digestion was performed by adding GluC and Trypsin directly into the prepared samples (two aliquots for each enzyme) and then the sample was incubated at 37 °C for 18 h. The digestion was stopped adding 5 µL of 100% formic acid to each sample. Sample purification was obtained with the SPE OASIS (Waters, Wilmslow, UK) treatment: activation (ACN), conditioning (1% FA + 1 mM DTT), sample loading, sample wash (1% FA + 1 mM DTT), and elution (60% ACN + 0.1% FA). The samples were evaporated to dryness in SPE-Dry and resuspended in 0.1% FA.

## 3. Results

One of the main analytical challenges in the field of vaccines research and development is the determination of the purity and quantification of the antigen in the final product of adjuvanted vaccines. The analytical tools commonly applied to characterize antigens in the adjuvanted vaccines require recovering them completely from adjuvant surfaces through desorption. The high concentration of salts and surfactants used in the desorption procedure could be involved in the loss of some chemical and physical properties that compromise its integrity. For this reason, there is a great need for tools that can directly quantify adsorbed antigens.

In the first part of this study, CE was used to simultaneously characterize multiple aluminum-adsorbed antigens in the final formulation. The approach to directly analyze antigens without any physical separation from the adjuvant has been explored with the potential to be widely applied to different targets (antigens) and for different formulations containing aluminum salts. Through CE, mixtures of nanoparticles (NPs) of different sizes can be separated, making this application suitable for the purpose according to the surface charges of alum and its colloidal particles [21]. In addition, CE has been shown in several reports to be able to quantify antigens with or without desorbing process from adjuvants [22,23,24]. The first attempt was performed to assess the ability of the assay to detect each of the adjuvanted vaccine components in a single run. A screening of different BGE (background electrolyte) solutions was performed to define which BGE caused the least interference during the run. The pH of the BGE was also optimized according to the isoelectric point of the analytes (see Section 2 for details) to ensure their migration within the capillary (complete protein protonation). The three recombinant proteins, fHbp, NadA, and NHBA, were firstly analyzed without alum as a mock run. 

Good detectability and resolution were obtained using a MEKC (micellar electrokinetic chromatography) modality with SDS as a surfactant. To expand the applicability to aluminum hydroxide complexes, the NHBA protein was selected as a case study. A titration experiment was performed by keeping the amount of NHBA fixed at 0.3 mg/mL while increasing the concentration of alum from 0.1 to 0.5 mg/mL.

The correct peak area (measured as the ratio between the peak area and its migration time) was monitored to assess whether the presence of alum had an impact on the antigen peak. In addition, the corrected peak area of the non-adsorbed NHBA is directly correlated to its amount (quantitative measure).

**Figure 3:**
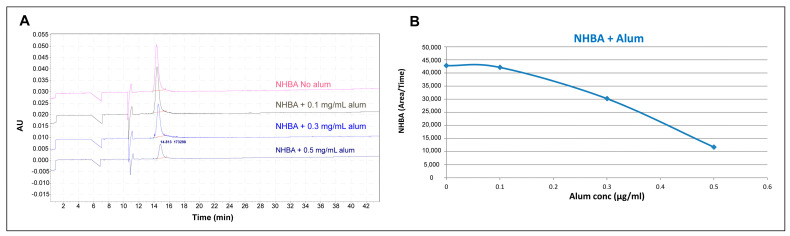
Electropherogram of NHBA with alum at different concentrations: (**A**): Recombinant protein was diluted at 0.3 mg/mL in ultra-purified water and aluminum hydroxide was added at a proper volume to achieve the desired final concentration of 0.1, 0.3, 0.5 mg/mL. Analytical method was performed with neutral capillary, length tot. 50.2 cm, id. 50 µm; BGE: Tris Acetate 100 mM pH 7.5 + SDS 15 mM; wash at 50 psi for 5 min with water, at 50 psi for 6 min with BGE. Autosampler temperature at 15 °C. Injection by pressure at 0.5 psi for 60.0 s. Separation at 12.0 kV (reverse polarity) + 0.2 psi. (**B**): Correct area of the NHBA in the presence of alum at increasing concentrations: the graph shows a decrease in corrected area of the protein, but it is not possible to follow the adsorption process on alum.

The preliminary results (Figure 3) demonstrate a correlation between the increase in concentration of alum and the decrease in the antigen corrected peak area. These data confirm that it would be possible to monitor the non-adsorbed portion of NHBA protein in the presence of alum. Under these conditions, however, no peaks related to free alum or alum-adsorbed antigen were observed. In addition, when applying the method to more complex matrices, as for example the other two model systems (containing the proteins fHbp and NadA), fHbp was not observed under these conditions. This is due to the positive net charge of fHbp at the pH of the selected BGE solution, which is suggested to create an electrostatic force in the capillary, thus resulting in a loss of migration of the protein in the capillary and subsequently no peak in the resulting electropherogram.

For this purpose, according to recommendations reported by Hoiczyk et al. [25], a screening of different BGE solutions were therefore performed to define which buffer caused the least interference during the run. The following BGE factors were considered and selected:

▪ TRIS Acetate pH 8.0
▪ Molarity of TRIS Acetate buffer (50 mM)
▪ SDS concentration (15 mM)

**Figure 4:**
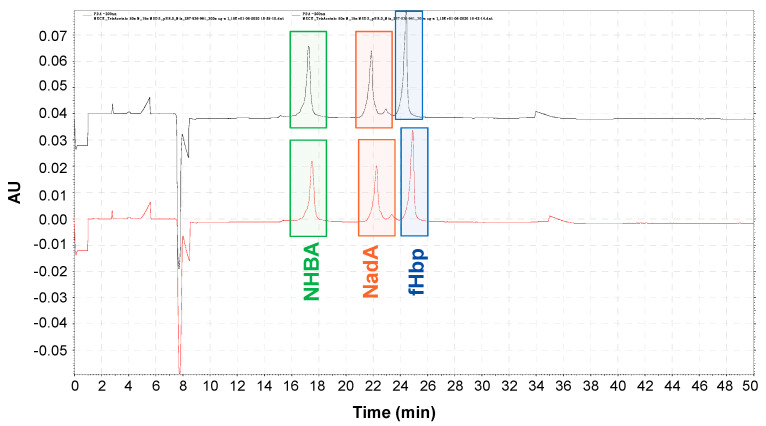
Electropherogram profiles of fHbp (blue square); NHBA (green square); NadA (orange square): three proteins were mixed in ultra-pure water to reach a final concentration of 0.1 mg/mL each. Separation method: Bare fused silica capillary, length tot. 70.2 cm, id. 50 µm; BGE: Tris Acetate 50 mM pH 8 + SDS 15 mM; 10 psi 3 min with water, 10 psi 3 min with HCl 0.1 M, 10 psi 4 min NaOH 0.1 M, 10.0 psi BGE. Autosampler temperature at 15 °C. Injection by pressure 0.5 psi 0.15 s. Separation 15.0 kV 40 min. The samples were injected in duplicate (black line first replicate; red line second replicate). Method shows good separation reproducibility and resolution between the three antigens mixed together.

The optimization of the assay was carried out by analyzing the combination of the three selected recombinant proteins in the same formulation (NHBA, NadA, and fHbP). The three recombinant proteins were mixed at 0.1 mg/mL each, without aluminum. Under the new electrophoretic conditions, a good resolution was obtained, and all the three recombinant proteins were detected with a good peak shape (Figure 4).

Following these preliminary results, the rate of adsorption of the three proteins was evaluated. The three recombinant proteins were treated individually under the same conditions. Their concentration was maintained at 0.1 mg/mL while the alum concentration was increased from 0.3 up to 3.0 mg/mL. The corrected peak area was used to monitor both the free antigen and aluminum–antigen complexes.

By evaluating the rate of adsorption of the three case-study protein antigens, initially one by one, it was observed that with an increasing concentration of alum-OH, the level of adsorption stabilizes for two out of three proteins (fHbp and NadA) between 1 and 2 mg/mL (blue line). This was also confirmed by monitoring the corrected area of the “adsorbed protein peak”, which increases as the corrected peak area of non-adsorbed antigen decreases.

**Figure 5:**
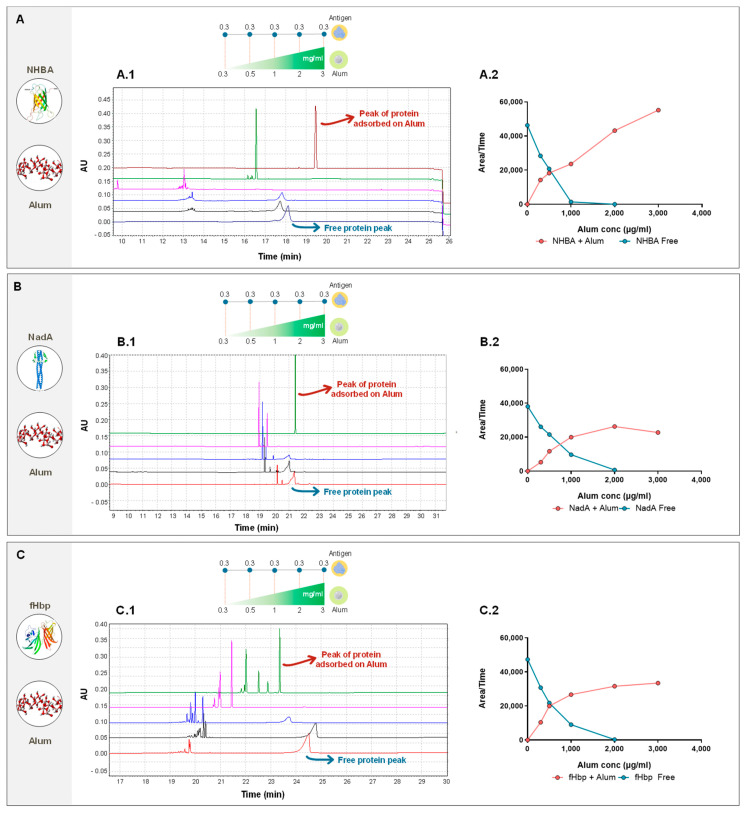
(**A**) A.1 The electropherogram profiles on NHBA, with alum at different concentrations (from bottom to the top: no alum, alum 0.3, 0.5, 1, 2, 3 mg/mL), A.2 The trend of corrected areas in function of alum concentration. (**B**) B.1 The electropherogram profiles on NadA, with alum at different concentrations (from bottom to the top: alum 0.3, 0.5, 1, 2, 3 mg/mL), B.2 The trend of corrected areas in function of alum concentration. (**C**) C.1 The electropherogram profiles on fHbp, with alum at different concentrations (from bottom to the top: alum 0.3, 0.5, 1, 2, 3 mg/mL), C.2 The trend of corrected areas in function of alum concentration. To evaluate the trend of corrected areas of each antigen, where multiple signals are present, as the value, the sum of all signals observed was considered.

On the other side, NHBA exhibited a distinctive behavior: while the free not-adsorbed protein peak also in this case disappears between 1 and 2 mg/mL (blue line), the signal related to the adsorbed protein keeps increasing without reaching a plateau (Figure 5).

This assay is able to detect both adsorbed and not-adsorbed proteins with a peak that is specific for each protein that can be easily integrated. The heterogeneous composition of the target matrix results in poor reproducibility of the electropherogram, especially for the alum-adsorbed samples, principally due to the presence of multiple sharp peaks (especially in the fHbp samples) at the highest alum concentration. The main cause of the multiple peaks was identified as the inhomogeneity of sampling due to the sedimentation of alum within the autosampler vial. The regular CE methods involved conditioning of the capillary prior to sample injection, and during this time the alum-adsorbed samples began their sedimentation process. This process leads to several alum populations with differing charges and sizes, thus creating inhomogeneous samples. Attempts to address this poor reproducibility were carried out, such as by injecting the sample immediately prior to capillary reconditioning to reduce the dead time in the autosampler vial during capillary rinsing, maintaining solution homogeneity. In addition, at the end of each run, the methanol wash was replaced with a HCl wash to reduce possible interactions between the capillary wall and the antigens. These changes helped to increase reproducibility; however, especially for multiple sample analysis, their application remained limited and not easily applicable for routine testing (e.g., in quality control analysis). Nevertheless, these results raise several questions around the behavior of NHBA in the presence of aluminum hydroxide. In particular, the fact that at such a high concentration of alum (3 mg/mL) the alum-adsorbed peak continues to increase in intensity suggests that there is a certain “conformational behavior” that is controlled by the amount of adjuvant.

For this reason, the behavior of NHBA was further investigated via alternative techniques to gain more insight into protein structure and how this relates to its potency. To study the alum–antigen interaction, multivalent (NHBA, recombinant fHbp, and NadA proteins) and monovalent (NHBA antigen only) formulations were tested in the presence of different alum concentrations. In order to assess whether the presence of alum at different concentrations might have an impact on antigen immunogenicity, a multivalent IVRP-Luminex instrument was used.

Immunological assays are currently widely used to monitor antigen potency content [26]. Indeed, while the immune response of alum is well documented, its effect on antigen stability remains under discussion. Protein stability can be affected by structural changes, such as protein unfolding, which occurs after adsorption to aluminum adjuvant particles. These effects are time-dependent, but it is unclear whether they are reversible or irreversible and whether the effects lead to a detrimental effect on immunogenicity [27,28].

IVRP has become a reference test to monitor vaccine potency and has been widely adopted and implemented as a commercial product’s control strategy that also allows animal-free testing.

The IVRP assay is a multiplex-based assay that relies on monoclonal antibodies directed against specific epitopes in the formulated vaccine. After the initial incubation phase, in which the mAbs recognize the specific epitope on the antigen, the free mAbs that do not react with the vaccine are isolated via centrifugation and quantified by using an indirect assay. The relative potency is measured using a parallel line model in which the inhibition curve obtained with respect to a reference batch with known potency (test batch) is compared to the sample.

One of the main advantages of this test in this context is its ability to work directly on formulated vaccines without being affected by the presence of alum (no sample desorption required) using a small quantity of material. Thus, insight into the native structural conformation of the antigen is possible in reduced time and with less experiments required.

**Figure 6:**
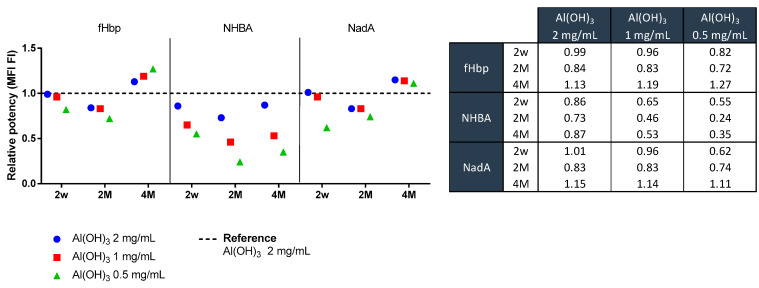
Representation of relative potency (RP) data obtained at three different time points for multivalent formulations (fHbp; NHBA; NadA) at different alum concentrations. The data were divided for each antigen at the different time points (2 weeks, 2 months, 4 months). The formulations at alum concentrations (2.0, 1.0, 0.5 mg/mL) were tested against the formulation at 3.0 mg/mL. The reference as such has an RP = 1 represented in the graph with a dotted line. Table reports the RP values obtained for each antigen.

The same experiment performed in CE was therefore performed by using IVRP. As the reference sample, according to the specific analysis, a mono-formulated or a multi-formulated sample with an alum concentration set at the maximum concentration level (3 mg/mL) was used. The IVRP data confirm that while for fHbp and NadA the potency equal to the reference is obtained at 1 mg/mL of alum (according to assay variability), the same behavior is not observed for NHBA (Figure 6). The IVRP results show the peculiar behavior of the NHBA antigen in both multivalent and monovalent formulations. A lower relative potency was observed as the concentration of alum decreased. This suggests that the lower quantity of alum reduces the ability of mAbs to bind antigen-specific epitopes. The results obtained over time (two and four months), moreover, suggest that the aging effect was not time-dependent. Furthermore, the percentage of adsorbed antigen did not influence the assay, as both free and adsorbed antigen were identified in the early phase of the assay. All these data suggest that, at lower alum concentrations, the surface area available for antigen adsorption is decreased. This may be related to the ability of proteins to change their structure. Indeed, under these conditions, proteins were able to change their structure to maximize adsorption. In addition, the decreased surface area means that proteins are adsorbed closer together, causing a masking of the sites involved in antibody binding. This effect was most evident in NHBA antigens, possibly due to the presence of the epitope in the Arg-rich region located in the flexible loop between the beta-barrel of the C-terminus and the N-terminus region [29,30].

**Figure 7:**
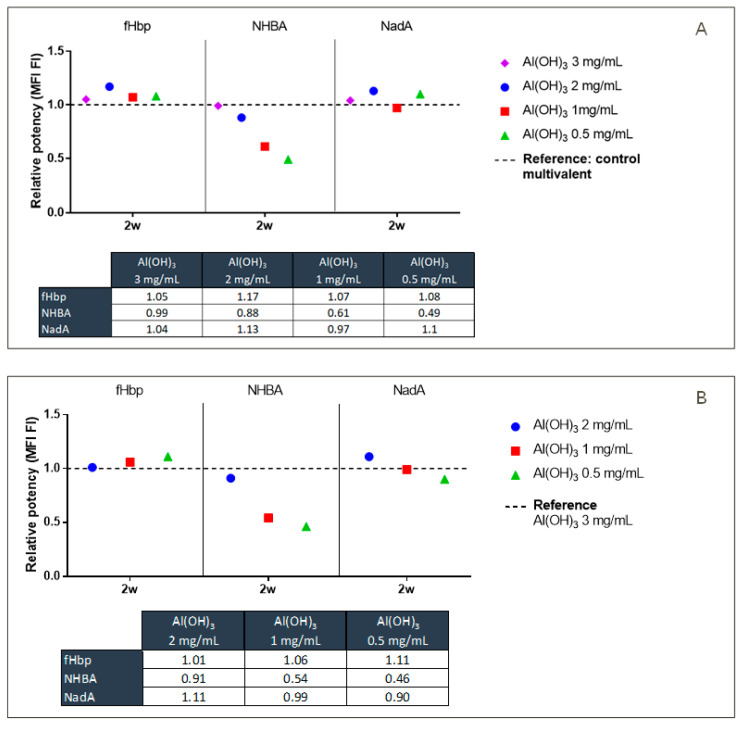
(**A**) Relative potency (RP) for freshly multivalent formulations (fHbp; NHBA; NadA) at different alum concentrations. The relative potencies for each antigen in the presence of different alum concentrations (3, 2, 1, 0.5 mg/mL) were compared to the RP = 1 reference old multivalent formulate at 3.0 mg/mL. The data shown as the freshly 3.0 mg/mL formulate presents the same potency of the old. Table reports the RP values obtained for each antigen. (**B**) Relative potency (RP) for freshly multivalent formulations (fHbp; NHBA; NadA) at different alum concentrations. The relative potency for each antigen in the presence of different alum concentrations (2, 1, 0.5 mg/mL) were compared to the RP = 1 reference freshly multivalent formulate at 3.0 mg/mL. Table reports the RP values obtained for each antigen.

This behavior was confirmed by formulating and adsorbing the three antigens simultaneous. To have more control within the analytical panel, a fresh multivalent formulation at 3.0 mg/mL of alum and 0.1 mg/mL for each recombinant protein (NHBA; fHbp; NadA) was also prepared. When the formulations at 3.0 mg/mL were compared, no differences were observed. NHBA seems to reach the target level of potency only at the highest concentrations of alum (>2 mg/mL) while being already fully adsorbed (Figure 7). As the assay does not discriminate between the percentage of antigen adsorption, this was taken to suggest a possible “rearrangement” of the protein to maximize its adhesion to alum when present at low concentrations. This may be related to the conformational propensity of NHBA, which might be strictly linked to its adhesion to the alum surface: the charge of alum may stimulate the formation of non-covalent bindings that may lead or drive to a specific structural conformation.

To further dig into the alum-mediated conformation of NHBA, additional MS characterization was performed. In particular, the hydroxyl radical footprinting (HRF) technique was applied to better elucidate the NHBA epitope in the presence and in the absence of alum.

HRF is a labeling approach to probe the solvent accessibility of residues within folded proteins through their covalent modifications.

The first demonstration of this technique was reported by Tullius et al. [31], who introduced Fe(II)-EDTA Fenton–Haber–Weiss chemistry to generate radicals from covalently modified macromolecules. The radiolysis chemistry of available amino acids increased exponentially over the years until Hanai et al. optimized the first method of protein footprinting via chemical modification in 1994 [32,33]. The common approach of HRF techniques function for determination of the residual solvent accessibility of protein side chains by irreversible covalent labeling [34,35] and to provide information on the identity and amount of the analyte in combination with liquid chromatography-MS (LC-MS) and enzymatic digestion. The hydroxyl radicals generated during protein footprinting experiments have several advantages over chemical reagents. First, water-like size allows them to penetrate all solvent-accessible sites. Second, their high reactivity can modify many amino acid side chains and their chemical selectivity is well understood. Third, they can be generated safely and conveniently under a wide range of solution conditions. All this makes them excellent probes for structural studies.

**Figure 8:**
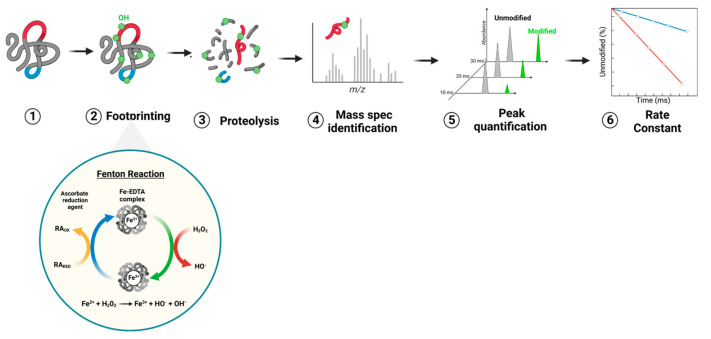
Schematic of an HRF experiment for rate determination. (1) Illustration of a generic protein, where residues on a protein in red are exposed to solvent and more prone to HRF, whereas other residues, in blue, are buried and less exposed due to tight packing and contact formation. (2) Covalent labeling of protein sites by hydroxyl radicals (green dot) that are generated from Fenton chemistry. (3) The enzymatic-digestion-broken protein in small peptide segments, cleaved by a specific protease. (4 and 5) Sequence and site of modified peptides are identified and the amount of modification is quantified based on tandem mass spectroscopy analyses. (6) A characteristic footprinting rate is determined for each peptide/residue segment based on a slope of oxidation rate as a function of exposure time (adapted from [37]).

Fenton Fe(II)-EDTA chemistry is the most commonly accessible method for generating hydroxyl radicals. In a Fenton-type reaction, hydroxyl radicals are generated through the oxidation of Fe(II) to Fe(III) by H_2_O_2_. The Fenton system (Figure 8) includes three essential components: Fe(II)-EDTA, H_2_O_2_, and ascorbate [36].

The reaction was monitored at multiple time points to determine the kinetics of modification of the amino acids susceptible to oxidation. The rate and modification of amino acid was found to be dependent on their side chain reactivity and on the ability to access different protein domains. The extent of the modification of the amino acid side chains in proteins in solution depends both on their ability to react with hydroxyl radicals and on their steric accessibility to the solvent. Methionine is one of the most sensitive residues towards oxidation, and its use guarantees the minimum oxidative stress for the conformational characterization of proteins to be determined. Each residue monitored by HRF required verification of the reliability of the response to oxidation; this acceptance criterion was satisfied when the quantity of oxidized residues is linearly proportional to the oxidation time.

Using this technique, it was possible to characterize the modified (oxidized) amino acids without desorption, thus giving a more realistic picture of the antigen structure as compared to a desorbed antigen.

**Figure 9:**
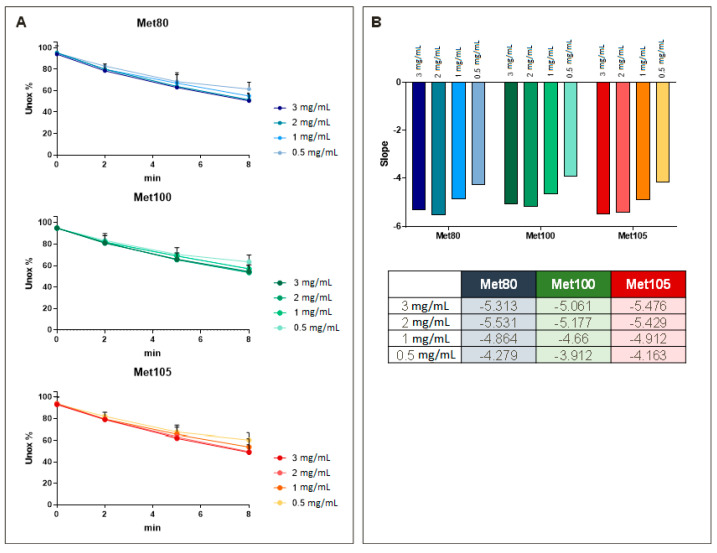
Comparison of NHBA formulations at different alum concentrations (3.0, 2.0, 1.0, 0.5 mg/mL). (**A**) Details of the oxidation rate of the three methionine residues 80, 100, and 105 are shown at the top as linear regression plots. (**B**) The slopes of the oxidation rate of the methionine residues are represented in barrel plots in the lower part, where the numerical values of the linear slopes for the three methionine residues are shown. A higher value corresponds to greater accessibility to the solvent.

The level of oxidation at five different time points (0, 2, 4, 6, 8 min) of the three Methionine residues (Met 80-100-105), already studied and known to be part of the epitope recognized by the monoclonal antibody (IVRP), were monitored. The results obtained (Figure 9) show that compounds with higher alum amount >2 mg/mL were oxidized to a greater degree than the samples containing lower concentrations of alum. This means that at lower concentrations of alum, the residues are less exposed to the solvent and therefore more masked. Consequently, when the immunoassay was performed, this resulted in a lesser exposure of the antibody to the epitope, confirming what was seen in the immunoassay experiments.

## 4. Discussion

In this work, we report our use of three different analytical tools to quantify detect and study the behavior of different antigens in the presence of the aluminum adjuvant. CE was utilized to separate, detect, and quantify the antigenic components in samples with complex matrices (representative of vaccine adjuvant formulations), and it may be used for the quantification of non-adsorbed proteins in vaccine samples without prior desorbing. CE was used to detect differences in quantification after the adsorption phase. Nevertheless, under these conditions, the presence of aluminum particles did not allow for the quantification of antigens adsorbed to alum, nor the identification of the single antigen–alum populations within a multivalent vaccine. To increase the efficiency of the technique, a resolution was used to reduce the sedimentation or to slow it down over time. Another possible solution could be the addition of chemical stabilizers (acting as anticoagulants) to the samples. Such agents could cause changes to the chemical and physical characteristics of the compounds, therefore alternating antigen–adjuvant interactions. An alternative, which perhaps would not alter the characteristics of the final product, would be to keep the samples in constant agitation until the point of injection. This would lead to greater poly-dispersion of the alum, with less chance of creating aggregates and thus settling over time. This configuration is not currently possible but may be investigated in the future. Nevertheless, NHBA showed distinct behavior with respect to the other antigens in CE. This led to further investigations via IVRP and HRF that showed that the presence of a certain concentration of alum is necessary to facilitate the correct conformation of NHBA to be recognized by monoclonal antibodies. Indeed, the comparative study of monovalent (NHBA) and three multivalent (recombinant proteins) formulations with alum through the IVRP assay and the HRF mass spectrometry confirmed the peculiar behavior of NHBA at different alum concentrations, in particular for monovalent formulations. Both assays demonstrated that in the presence of low concentrations of alum, the protein tends to change its conformation to maximize adhesion, probably leading it to assume a spatial conformation in which epitopes are less accessible to the antibodies used in the IVRP assay.

## 5. Conclusions

The analytical synergy of these three techniques developed and used in this work allowed us to gain a comprehensive understanding of the binding of different antigens to alum. In detail, the CE method can be applied to monitor changes in the amount of antigen derived from the adsorption process. The combination of physiochemical characterization and immunological studies (HRF and IVRP), as orthogonal assays, allowed us to characterize in depth the conformation changes of the antigen in the presence of adjuvants. Indeed, the synergy of these two techniques is shown as the three antigens compete to maximize the adhesion of the adjuvants at lower alum concentrations. It is to be noted that all the techniques used here to characterize the adsorption/structural features of the antigens when adsorbed to alum do not require any sample pre-treatment, avoiding any bias that might be related to the desorbing steps.

## 6. Patents

F.B. is a named inventor on patents on related topics.

## Figures and Tables

**Table 1:** mAb selected for IVRP assay.

Antigen	mAb Clone	Isotype	Source Purity	Epitope Recognized
fHbp	12C1/D7	IgG2b	Hybridoma purified	Conformational
NadA	6E3/29	IgG1	Hybridoma purified	Conformational
NHBA	10E8/A5	IgG2b	Hybridoma purified	Conformational

## Data Availability

Data are contained within the article and supplementary materials.

## Supplementary Materials

The following supporting information can be downloaded at: https://www.mdpi.com/article/10.3390/pharmaceutics16030420/s1. Figure S1: New analytical tools with the aim of directly quantifying and assess both the content and/or the purity of formulated alum-adsorbed antigens, without any preliminary sample manipulation (e.g., antigen desorption). The different conformation / behaviour in terms of response to specific mAbs in presence of different ratio of alum-OH adsorbent antigens have been investigated. As a proxy to develop new analytical tools, three model adsorbed recombinant proteins were used: NadA, NHBA, fHbp as antigens and alum-OH as an adjuvant system. The selection of adjuvanted system model was dictated by the substantial quantity of literature regarding the proteins structure and immunological activities, meaning that they are well characterized, including their adhesion rate to aluminum.
