# Supplementary material for: Analytical Insights into Protein–Alum Interactions and Their Impact on Conformational Epitope
Source: Pharmaceutics. 2024 Mar 19;16(3):420. doi: 10.3390/pharmaceutics16030420 (PMC10975739; doi:10.3390/pharmaceutics16030420)

SUPPLEMENTARY MATERIAL

Figure S1. New analytical tools with the aim of directly quantifying and assess both the content and/or the purity of formulated alum-adsorbed antigens, without any preliminary sample manipulation (e.g., antigen desorption). The different conformation / behaviour in terms of response to specific mAbs in presence of different ratio of alum-OH adsorbent antigens have been investigated. As a proxy to develop new analytical tools, three model adsorbed recombinant proteins were used: NadA, NHBA, fHbp as antigens and alum-OH as an adjuvant system. The selection of adjuvanted system model was dictated by the substantial quantity of literature regarding the proteins structure and immunological activities, meaning that they are well characterized, including their adhesion rate to aluminum.

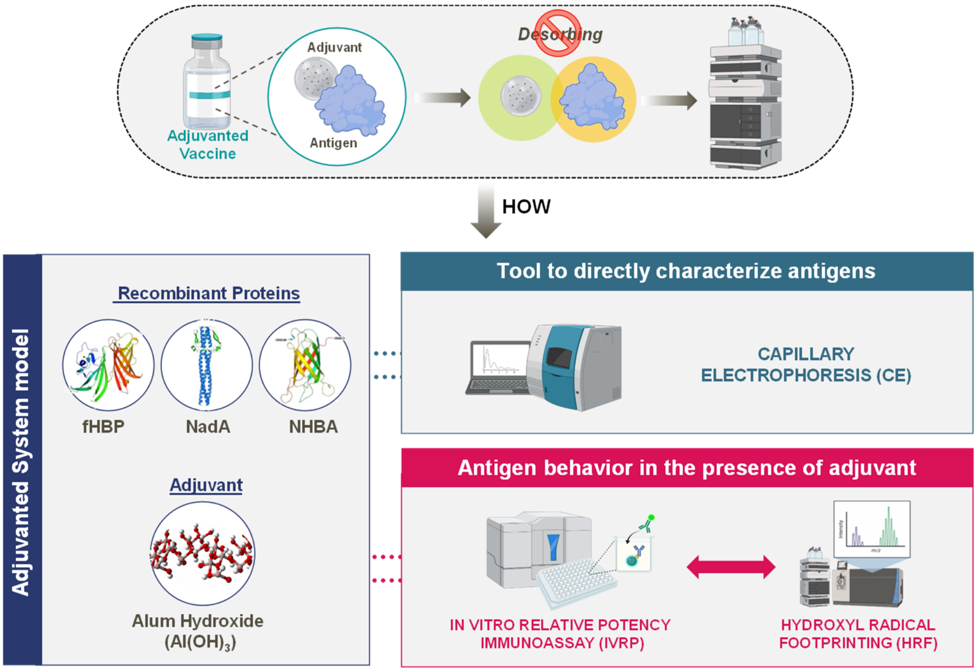

Supplement: Supplementary file 1 [file pharmaceutics-16-00420-s001.zip › pharmaceutics-2866654-supplementary.pdf]
